# Is Collaborative Care the Future of Medicine? Lessons Learned from the Care of Children with Colorectal Conditions

**DOI:** 10.3390/children11050570

**Published:** 2024-05-09

**Authors:** Julie M. Choueiki, Stephen Sales, Susan Callicott, David Ashman, Katherine Worst, Andrea Badillo, Briony K. Varda, Allison Mayhew, Thomas O. Xu, Marc A. Levitt

**Affiliations:** Division of Colorectal and Pelvic Reconstruction, Children’s National Hospital, Washington, DC 20010, USA; jchoueiki@childrensnational.org (J.M.C.); ssales@childrensnational.org (S.S.); scallico@childrensnational.org (S.C.); dashman@childrensnational.org (D.A.); kworst@childrensnational.org (K.W.); abadillo@childrensnational.org (A.B.); bvarda@childrensnational.org (B.K.V.); toxu@childrensnational.org (T.O.X.)

**Keywords:** pediatric colorectal, Hirschsprung disease, functional constipation, multi-disciplinary care, anorectal malformations, collaboration

## Abstract

The treatment of patients with colorectal disorders requires care from a wide variety of medical and surgical specialties over the course of their lifetime. This is ideally handled by a collaborative center which facilitates the assessment and development of patient care among multiple specialties which can enhance the quality and implementation of treatment plans, improve communication among different specialties, decrease morbidity, and improve patient satisfaction and outcomes. This collaborative approach can serve as a model for other parts of medicine requiring a similar multi-disciplinary and integrated method of care delivery. We describe the process, as well as the lessons learned in developing such a program.

## 1. Introduction

When someone uses the word “pelvis”, what comes to mind? From an anatomic point of view, the structures contained within include the colorectal, urologic, gynecologic, and gastrointestinal systems. Each system is packed in a tight contiguous anatomic space, bound by the bony pelvis; therefore, the physiologic functions of one system are intimately associated with other systems. Several specialties are dedicated to the unique care of each of these systems (colorectal surgeons, urologists, gynecologists, gastroenterologists), however, despite the intimacy of their respective structures within the pelvis, these specialists commonly work in a silo, independent from each other. Attempts to collaborate are typically few. Through years of experience collectively taking care of children with disorders of the pelvis, it has become obvious that a collaborative model should be applied.

Outside of medicine, many fields utilize a collaborative approach by design. Imagine a project for building a new house. How does such a project get off the ground, literally? Does the bricklayer start to build the walls before the foundation is set? Does the electrician come and wire the house before the framing is done? In practice, all involved participants would likely meet to discuss the plan, coordinated by some sort of project manager (an architect perhaps) who helps delineate each partners’ responsibilities.

For complex patients, why do we not employ a similar collaborative method? How often does a urologist contemplate performing a bladder augmentation or ureteral reimplant in a patient without considering the impact a constipated colon may be having on the child’s bladder/renal function? How frequently does a patient undergo the creation of an antegrade continence enema utilizing the patient’s appendix without considering a patient’s bladder function and/or the need for a continent catheterizable channel for urine? Just how a good plumber should consider how their electrician colleague may want to complete their wiring before telling the builders to cover the frame in insulation and drywall, the colorectal surgeon should take into consideration their fellow urologist, gynecologist, or gastroenterologist. If it is anything like the house building analogy, the care of children with complex disorders of the pelvis should have input from everyone prior to proceeding with an intricate plan.

Mandatory reading in this field should include the children’s book *Everyone Poops*, by Taro Gomi [1], which explains how important the physiology and mechanics of stooling are in a child’s early development, something that is often taken for granted. Most parents with newborns diagnosed with a colorectal problem never seem to have considered that their child could have a problem with the emptying of their bowels. When encountered with a child with a colorectal problem, parents quickly focus on whether the surgery indicated will recreate “normal” anatomy and, most importantly, allow their child to stool without difficulty or embarrassing accidents so that they live a life all parents wish for their child—a life unburdened by poor health. As providers of complex care, it is important to remember what goals and wishes the family and patient want, and to strive to achieve those goals. Ultimately, it is the functional outcome that matters most.

These concerns are not new, as children with colorectal problems can be traced back thousands of years. There is a 2000-year-old reference in the *Babylonian Talmud*, written in 200 C.E., recommending that “an infant whose anus is not visible should be rubbed with oil and stood in the sun…and where it shows transparent the area should be torn crosswise with a barley grain” [2]. Surgical techniques to manage such a patient have certainly evolved since that time, but the basic tenets of care remain unchanged.

An experience in the clinic demonstrates the challenges we face but also what we can accomplish in this field. A six-year-old girl with a history of a newborn repair of an anorectal malformation presented with concerns for soiling. She was miserable, citing significant teasing and distress at school. The team detailed to her that after a planned bowel management week she should be clean of soiling and able to wear regular underwear. She gave the team that quizzical, doubtful look children often give to adults, like they have no clue. One week later, after implementing a successful enema program, the child entered the clinic again, now with a big smile and said, “You guys make good promises!” It is that moment, the culmination of complex care into a desired clinical result, that makes all the effort worth it.

## 2. The History

A review of the history of pediatric colorectal surgery may help set the context. The senior author of this paper (MAL) was an eager medical student in 1992 and took an elective with Alberto Peña, one of the pioneers in the field of colorectal care, which began for him a 30-year journey. A key memory from that time was that Peña provided all aspects of care for a complex group of patients. To his patients, he was not just the colorectal surgeon. He was the urologist, gynecologist, psychologist, and even the social worker. However, with medicine becoming increasingly complex, it became clear that this was not the job of one person. Much like the architect designing a house, it takes a village of specialists to help care for these children. Other fields, such as transplantation, neonatal care, and cardiology, were already benefiting from a collaborative approach at this time. Colorectal surgery as a specialty needed to quickly catch up and become a multi-disciplinary field with help from key collaborators.

**Figure 1:**
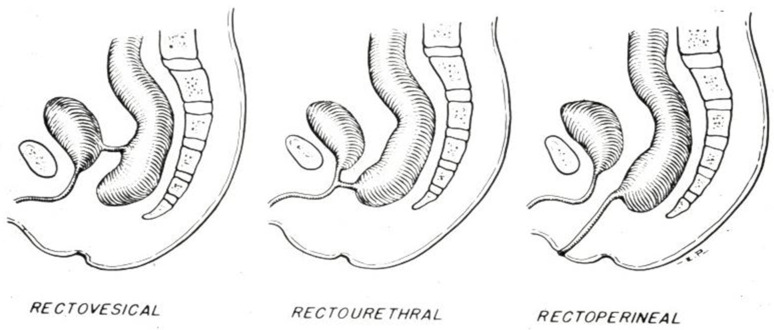
Figures of anorectal malformations from the original Gross and Ladd textbook 1942 [4].

The modern field of pediatric colorectal surgery began in the 1940s in Melbourne, Australia. Frank Douglas Stephens, who trained at Great Ormond Street Hospital in London, worked on defining the anatomy of children with anorectal malformations by performing autopsies on 12 patients who had died with these malformations [3]. After Stephen’s publications it became clear that surgeons had, at that point in time, not really understood the true anatomy of such patients. Their concept was both oversimplified and inaccurate, and is seen illustrated in the images shown in Figure 1, in the textbook by Robert Gross [4].

**Figure 2:**
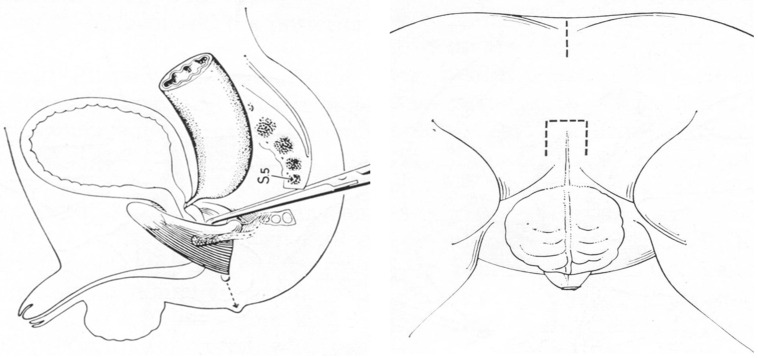
Figures from Stephen’s sacroperineal technique, 1953 [3].

Stephens learned that when the anus had failed to develop, the puborectalis muscle coalesced behind the urethra, and posteriorly there was no muscular anatomy of any significance. From these observations, he developed an operation that found, via a small perineal incision, a dissection plane posterior to the urethra and anterior to the puborectalis sling through which he could pull through the rectosigmoid colon and create a neoanus [Figure 2]. Not long after, William Kiesewetter in Pittsburgh performed such a pull-through using similar anatomic principles [5]. Both surgeons utilized a sacral approach to the pelvis, which they had learned from previously performed operations in adults [6].

Justin Kelly, a trainee of Stephen’s in Australia, learned how to perform this operation. He then received additional training at Boston Children’s Hospital in the late 1960s. While there, the surgeons he worked with wanted to learn about the new technique, and he taught them what he had learned. One of these surgeons was Peña, a surgeon from Mexico who had come to Boston to learn from Robert Gross, the chief of pediatric surgery there. At that same time, Peña was also influenced by Hardy Hendren, a master surgeon who was the forerunner in the care of cloacal malformations. Hendren worked at the Massachusetts General Hospital across town, and Peña and his fellow residents would go watch him operate.

**Figure 3:**
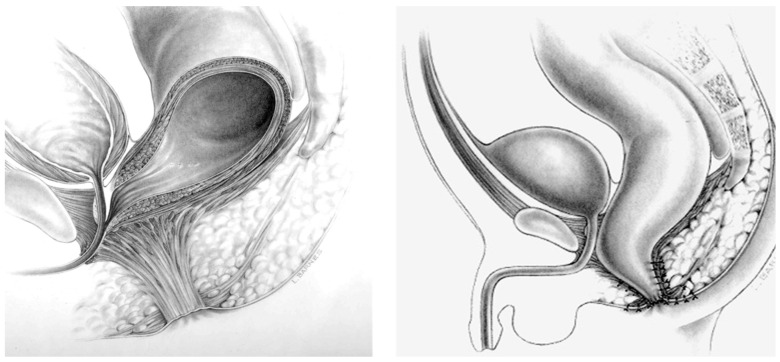
Diagrams of posterior sagittal anorectoplasty (PSARP) [5].

After his time in Boston, Peña moved to Mexico City to become the chief of Surgery at the National Institute of Pediatrics in 1972, at the age of 34. One of Pena’s first moves as chief was to ask his fellow surgeons to pick a field of specialization. Colorectal surgery was left unchosen, so Peña decided to take on that group of patients, thus catalyzing the start of his revolutionary career. Peña initially utilized Stephen’s technique to repair anorectal malformations, but he quickly became frustrated with the approach as the maneuvers were blind and provided very poor exposure to the distal rectum. Over time, he lengthened the incision, which led to a full-length posterior sagittal incision from the distal sacrum to the perineum. At the time, this approach was controversial because such an incision “cut the sphincters”, which were thought to exist in a circle. Pena demonstrated, consistent with Stephen’s observations, that the sphincters are more like a funnel, and if they are divided perfectly in the midline and then reconstructed after rectal mobilization, the muscles can still function well. Thanks to a collaboration with Peter Devries, who came to Mexico City to work on these cases with Peña, the posterior sagittal anorectoplasty was first presented at the Pacific Association of Pediatric Surgery meeting in 1980 [Figure 3] [7].

This approach to patients born with an anorectal malformation allowed a view and an understanding, for the first time, of the true pelvic anatomy. Seeing the anatomy from this approach allowed surgeons to solve problems that, to use Peña’s words, were “too difficult to reach from above (via laparotomy) and too far to reach from below (via a perineal approach)”. This new anatomic understanding was thereafter used for the repair of cloacal malformations [8], urogenital sinus malformations [9], pelvic tumors [10], urethral pathology [11], reoperations for anorectal malformation [12] and Hirschsprung disease (HD) [13], a transpubic approach for complex genitourinary problems [14], and to manage cloacal exstrophy [15].

Just as important as these surgical innovations was Peña’s impact on the postoperative care and quality of life of these patients. He introduced a focused approach to bowel management for the treatment of fecal incontinence [16]. Peña realized that surgical correction was not the only aspect of care that these children required to ultimately achieve fecal continence. It was clear that just achieving a good anatomic reconstruction did not necessarily deliver a good functional result. Such bowel programs required experienced nurses and advanced practice providers to focus on following children through their social and physical development, supporting them with adjustments along the way in their diet, habits, laxatives, and/or mechanical enemas. Since he laid the groundwork for bowel management, these programs are now available at many centers world-wide and now thousands of children can have their stomas closed, are free of diapers, and wear regular underwear just as their peers do. This is comparable to the way certain patients with urologic problems have been made dry using intermittent catheterization for urine [17].

## 3. Collaborative Care Model

The collaborative care model is not unique and has already demonstrated to be successful in areas of pediatric surgery such as bariatric surgery, ECMO, fetal surgery, transplantation, trauma, and vascular anomalies. There is now documented value for such a model for care in biliary atresia [18], bladder exstrophy [19], sub-specialization in pediatric surgery [20], pediatric oncology [21], and rectal cancer [22]. This effort in the collaborative model is evidenced by the massive effort in the European Union to create European Reference Networks which aim to provide high-quality, cost-effective care by concentrating the knowledge and resources needed to treat rare conditions in centers of excellence [23].

How does an institution start the process of creating a multi-disciplinary colorectal program? The first step is to obtain a team comprising clinicians who are both skilled and passionate about helping these children. The problems of the complex patient are not quickly solved and require persistence and patience from the providers and clinicians. A strong work ethic and commitment to this field are critical. Next, knowledge of colorectal and pelvic anomalies is a unique and necessary skill. These skills are acquired through experience. Ample empirical studies exist demonstrating a positive relationship, for both surgeons and institutions, between the volume of complex surgical procedures performed and a decrease in the rate of complications, reoperations, readmissions, and postoperative length of stay.

Consider pediatric surgery training as an example of this idea. Trainees on average perform 10 esophageal atresia repairs. Once that trainee begins their career as a surgical consultant/attending, they likely will perform only one or two such cases per year; so, during a 30-year career, this amounts to approximately 30 to 40 cases. Now imagine if a new faculty member was provided with that same number of esophageal atresia repairs but asked to perform them all over the next two months. During that time, that surgeon benefits from the help of many experts and can make small, incremental changes to their technique. Will the 30th case in both scenarios undergo the same repair? Likely, the high-volume, concentrated experience would lead to small improvements in technique and a final case that is superior to the first one. It is this level of experience for complex surgical problems that is essential to developing new ideas and to improving outcomes. This concept is much akin to a famous quote by Bruce Lee, the martial arts expert, when he said “I fear not the man who has practiced 10,000 (different) kicks once, but I fear the man who has practiced one kick 10,000 times”. A surgeon’s capabilities are best optimized when they place a concentrated focus on a small set of problems rather than spreading their expertise thinly across many.

## 4. Programmatic and Financial Implications

One of the first aspects of establishing a collaborative program is to determine if the organization has both the capability and the capacity to deliver the care required by the new patient population. The clinical advancement of patient care is best aligned with the financial impact, either by bringing in money to the hospital or, depending on that hospital’s incentive system, by promoting cost savings with improved care, which is universal to any health system. Although this paper focuses on some of the financial implications centered in a USA-based healthcare system, the cost-saving principles discussed should benefit even readers from outside the United States.

Surgeons and physician leaders with significant experience with the clinical needs of new patients must identify the specific medical capabilities required to treat the myriad of clinical challenges in this population. Do these skills exist in the organization, or must they be recruited for? If they exist in the organization, do they exist in sufficient capacity to meet the added demand of the new collaborative center? The recruitment of additional professional staff could add 9–12 months to the expected start date of the new program. Hospital leaders will want to understand these patients’ impact on the organization. To answer that question requires much work, data analysis, and a few assumptions.

Some of the first questions to answer are the following: (1) What is the size of the market? (2) Who are the primary competitors? (3) What share of the market is likely to seek care in the new program? Such data are difficult to access. In the USA, one source is the Children’s Hospital Association’s Pediatric Health Information System (PHIS) database. The PHIS database contains de-identified data on the surgical procedures performed at member hospitals. The PHIS database can provide a reasonable estimate of the market for the procedures in question, identify the main competitor hospitals performing those procedures, and estimate the volume of those procedures performed at each hospital. Once an estimate of patient volume is completed, a budget and pro forma financial documents can be created to illustrate the expected revenue and expenses associated with the new center. In the USA, creating financial estimates requires some assumptions about reimbursement rates from insurance payers and Medicaid. Outside the USA, similar assumptions must be made to estimate the financial burden of creating a new program based on a country’s respective health system and payer structure.

Will creating a collaborative center increase office visits, surgical cases, and in-patient bed days? Or from a cost-saving perspective, will hospital admissions or emergency care be reduced? If the answer to these questions is yes, the follow-up question is perhaps the most important. Are there additional clinic blocks, operating room times, and inpatient beds available to accommodate the increased demand of these new patients? Will such patients generate additional radiology, motility, or urodynamic studies? Are these services available, and are they available at the time required by the cadence of the patient visit, which may include a variety of ancillary studies, a clinic visit, an exam under anesthesia, a reconstructive surgery, and several days of in-patient stay? Each component of a patient’s care is ideally choreographed precisely to minimize the length of the visit and the inconvenience to the family, particularly for those travelling from afar. And lastly, will providing unique care for a complex patient population bring additional revenue (or cost savings) to the hospital? The program needs to establish metrics to track the progress of these items, including clinical outcomes, quality improvement, patient satisfaction, staff satisfaction, and financial performance. Many of these reports, especially ones measuring the downstream workload financial impact of these patients on hospital services and revenue may not currently exist in the organization.

Will such an approach enhance the hospital’s reputation? Will the effort save costs by improving outcomes? Better quality of outcomes may lead to increased demand for care, similar to theme of the movie *Field of Dreams*, which noted that “if you build it, they will come”. For complex surgeries like cloacal repair, quality outcomes lead to increased volume, which, in turn, leads to further improvements in quality. Better quality brings reduced cost, most notably in fewer subsequent redo repair surgeries, shorter hospital stays, fewer patients needing renal transplantation, etc. Perhaps not during a specific episode of care, but over the lifetime of a patient with a complex medical condition, better-quality outcomes due to the superior experience of, and collaboration among, surgeons and physicians may lead to fewer tests, fewer inpatient days, fewer surgeries, and reduced total cost of care. From the family’s perspective, better-quality outcomes lead to fewer missed days of school, fewer missed social activities, and a higher quality of life for the patients and their families. Most likely, the quality outcomes of the center will enhance the reputation of both the hospital and the program, but the results must be known to the relevant patient population and to the insurance payers expected to pay for the care. This process begins with a well-designed plan for tracking data, which should be implemented before the first patient arrives to allow for a robust and valid database. Capturing relevant fields of productivity for the center will allow for data extraction to support arguments for additional resources as the program grows and facilitates research activities [24].

On day one, the marketing team needs to get the word out that a new specialized center is ready to see patients and provide multi-disciplinary care. Over time, robust research activity provides the source material for a successful marketing campaign. The center must demonstrate that its outcomes are indeed better than those available elsewhere. These two activities, research, and marketing, go hand in hand. Patients, referring providers, and insurance company representatives read those articles. Sound empirical research demonstrating the quality of the center’s outcomes is the basis for leveraging those outcomes financially and growing the center.

Lastly, if the organization hopes to be paid for services provided in the USA, it is critical to ensure all licensed independent providers are enrolled with government payers such as Medicaid and private insurers, both in and out of state. A program should expect at least a four- to six-month lead in time to complete the process. Approvals and applications for Medicaid can range from 30 to 60 days for in-state applications and up to 3–6 months for out-of-state applications. To receive proper reimbursement for treatment rendered, the business team must work closely with the clinical team to ensure that providers and the hospital are credentialed with the payers and patients pre-authorized for specific services. Authorizations are often challenging to receive from out-of-state insurance carriers, especially state Medicaid agencies trying to keep their state funds at home. A dedicated team of insurance specialists can facilitate the pre-authorization process to ensure patients can access the center’s specialized care. This process may include coaching patients on how to request a referral to the center, working with primary care and specialty care physician offices on crafting a letter of medical necessity, providing research studies to state Medicaid agencies to demonstrate the value of care within the center, and arranging peer-to-peer conversations with insurance medical directors to speak to the center’s clinicians about the care provided. Finally, to mitigate a potentially cumbersome process, it is imperative to have a strong relationship with the hospital’s managed care department and revenue cycle personnel. Creating templates for standard processes, such as primary care referral templates, clinical letters of necessity by diagnosis, and other such documents customized to individual patient’s treatment plans, can help expedite the financial and administrative barriers to providing patient care.

The care team members are critical to establishing a successful multi-disciplinary team once the commitment and buy-in have been established, and one that will approach the child’s care holistically. An example of the full-time equivalents (FTEs) needed to set up a multi-disciplinary center are listed in the following Table 1:

Building the case for these resources will be progressive, so it is best to project a vision for increasing supply given the demand over time, which includes the downstream impact on ancillary services coupled with the anticipated length of inpatient stays [25].

Depending upon the center’s potential market and the dedicated personnel in the program, a reasonable first year can project 400 colorectal cases, 200 inpatients, and 200 outpatients with an average length of stay of five days. From the initial investment, the annual clinic visits are estimated at 800 for pre- and postoperative care. Depending upon the number of out-of-the-region patients, 90 percent of these patients can be considered in-person visits. Advanced practice providers holding bowel management clinics for the management of fecal soiling are a vital component of a comprehensive program. In our experience, one-third of the children participating in the bowel management program will need a surgical intervention; thus, such a nursing-led program is a driver of surgical volume. While the ancillary and downstream assumptions vary from program to program, the patients will require various radiology, lab, and motility studies to help with the treatment plan. The assumption beyond year one is an estimated 3% growth rate depending upon the expansion of the original multi-disciplinary program providers, diversification into transitional care, the recruitment of additional surgeons or providers, and the program’s reach beyond the identified market area.

Payment for specialized services comes from multiple sources, including the government, private payers, cash-based payment, and other insurers. Over the last ten years, the United States has had a significant number of changes in the reimbursement system. In 2010, the Patient Protection and Affordable Care Act (ACA) came into being. This included the most sweeping and expansive Medicaid services to date. New payment models include pay-for-service, bundled payments, and quality initiatives such as the hospital readmissions reduction program [26]. Other insurers or payers often follow the reimbursement structure outlined by the Center for Medicare and Medicaid Services. The clinicians must provide robust clinical templates that outline each inpatient admission’s acuity and specialized service needs. The diagnostic-related group (DRG) system categorizes inpatient admissions based on diagnoses and other factors. Medicaid and other insurers pay for the hospital stay based on severity and diagnoses. Standard operating procedures, outcome metrics, and quantifying the impact of a destination center such as colorectal surgery will impact sustainability and expansion.

## 5. Nursing Care

For patients with colorectal problems, including those with anorectal malformations (ARM), Hirschsprung disease, fecal incontinence (related to a variety of conditions), and colonic motility disorders, the collaborative care model is key, because such patients have multiple systems affected and are best served by a variety of specialists throughout their lives. These include providers in the fields of colorectal surgery, urology, gynecology, gastroenterology, motility, orthopedics, neurosurgery, anesthesia, pathology, radiology, psychology, social work, nutrition, pelvic floor physical therapy, and many others. To achieve a good functional result for a colorectal patient, a lot of postoperative tinkering of their care is needed. From the surgeon’s perspective, a complex colorectal operation takes about three to four hours to perform. From a holistic perspective, it takes hundreds of hours of additional work to achieve good results—the vast majority of which relies on good nursing care.

At many colorectal centers across the world, nurses are being utilized to improve patient care and outcomes from the moment a problem is identified until the patient is thriving postoperatively. Nurses are great at collecting information about a patient and truly understanding a family’s goals for treatment before any plan is set into motion. Their training makes the need to educate families on their child’s anatomy and the procedures being planned an inherent aspect. While surgeons are often limited in how much time they can spend with a family due to their operative room duties, a nurse is able to connect with patients for longer and reassure them that their understanding of the events to come is accurate. Well-trained nurses and advanced practice providers (APPs) are then poised during the surgical process and recovery to identify concerns and address them promptly and appropriately. Once a child has recovered from surgery, a nurse and APP can then aid a family through ongoing challenges such as skin care, diet, and bowel management.

Having well-trained nurses and APPs requires investment from the surgical department and hospital. These clinicians need to be versed in colorectal diagnosis, treatment options, and the benefits of collaboration. If a nurse understands a diagnosis as well as the operation that is required to offer the anatomic correction, they will be much better prepared to answer the family’s questions, provide anticipatory guidance, and act on a patient’s medical needs. While all nurses are trained in how to assess and triage patients as well as implement care, it is imperative that a surgical department educates its team so that they are prepared to care for these medically complex and chronic patients. Nurses are great problem solvers, are willing to engage the patient at the bedside, and are uniquely positioned to fill the gaps in care. Without superb nursing partners, surgeons caring for this patient population will achieve very little.

Bringing new patients to the collaborative program is critical to capturing the revenue needed to support the business model. One must plan for patient and family support that is the most cost-effective. Prioritizing the surgeon’s time performing surgeries is the best use of their expertise and allows the organization to maximize their time generating revenue for the fiscal health of the organization. The utilization of a strong nursing (RN) team and an advanced practice provider team (APNs and PAs) can facilitate the significant number of hours it will take to care for the complex patient outside of the operating room.

Investing in the education of these nurses, physician associates, and nurse practitioners should not be undervalued. When given proper education and training, the registered nurse can help save the organization money while providing exceptional customer service from the first contact with the family. When a new patient inquiry reaches out to the program, the well-trained nurse can connect with the family, gather, and analyze all relevant health records, ascertain the patient’s goals, as well as uncover any psychosocial needs that would facilitate the best outcome. Upon completion of this work, the nurse will be able to draft a proposed plan of care including testing, imaging, and surgical care. The nurse can then review the plan with the surgical, medical, and psychosocial teams. Having the information all presented in this manner, before the patient ever appears in the clinic, can save the team time. Additionally, the family has the best experience possible with the nurse serving as a navigator for their child, bringing all the surgical partners together at one time to review the child’s case and collaboratively plan. Families avoid the arduous task of reporting to multiple different clinics to see different specialty services. This same nurse is now intimately familiar with the child’s course of treatment and will be the first point of contact for the remainder of the child’s time with the program, triaging their needs and working to the fullest scope of their license to treat the patient.

Facilitating care even further is the advanced practice provider team. Made up of nurse practitioners and physician associates, these mid-level providers can see patients with autonomy, taking on the role of managing the long-term care and outcomes for the patient. Follow-up surgical and ongoing bowel management visits can be provided by the APP and, again, allow the surgeon to prioritize their time performing surgeries. The families benefit from the expertise the APP has developed in caring for this unique population in large volumes and being actively engaged in research in this specialized field.

While it becomes obvious to those on the collaborative team that nursing and advanced practice providers are necessary components for the best clinical outcomes and exceptional customer experience, it will not be uniformly evident to others within the organization. Colorectal care follows more of a chronic care model, meaning that patients keep coming back and do not drop off the radar as they may do in most other surgical programs. Colorectal expertise is required for many years, even into adulthood, and even after a successful surgery. It is imperative that the work these team members provide can easily be quantified. This can be achieved by tracking the number of telephone encounters, emails, portal messages, or APP clinic visits that have occurred. Demonstrating the time APPs spend with and care for these children will truly quantify how much time was alleviated from the workload of the surgeon, which not only facilitates the best possible patient experience, but is a major cost saving for the organization.

## 6. Benefits Experienced from the Collaborative Care Model

The multi-disciplinary approach has led to numerous advances in the field of colorectal and pelvic reconstruction. These innovations have improved the care and quality of life of many children over time. Some examples include the following:

### 6.1. Prenatal Diagnosis

Prenatal diagnosis of anorectal and cloacal malformations is a growing field. Now, clinicians assessing fetal imaging are more aware of certain constellations of findings that may indicate pre-natal colorectal diagnoses. For example, the presence of a single kidney and pelvic mass in a female fetus may indicate a cloaca. Additional attention and screening of clitoral anatomy, pubic bone diastasis, bowel dilation, sacral development, absence of the radius bone, as well as cardiac, spinal, and renal anomalies may all hint at the diagnosis of an anorectal malformation [27]. Prenatal diagnosis allows parents to meet specialists in advance of delivery and to become educated about the diagnosis and postnatal management. Early diagnosis also helps inform the delivery plan and direct neonatal management, minimizing the potential for complications and improving outcomes.

### 6.2. Newborn Management

With dramatic improvements in neonatal care, management of newborns has been revolutionized [28]. These have included advances in radiology—e.g., contrast studies of the colon for Hirschsprung disease, the diagnosis of hydronephrosis, 3D reconstruction of cloacal anomalies [29], ultrasound-guided distal colosonagraphy for anorectal malformations [30], as well as improved interventional and surgical techniques in the management of hydrocolpos and stoma creation.

### 6.3. Urologic Anomalies

It is vital to recognize associated urologic anomalies in patients with anorectal malformations. The rates of chronic renal disease and the need for bladder augmentation or renal transplantation are decreasing with improving urologic care and proactive bladder management [31,32]. Furthermore, increased collaboration with urologic providers allows for more coordinated care, potentially consolidating visits and/or operations that may have occurred separately in a patient who does not received care in a collaborative model.

### 6.4. Gynecologic Concerns

Understanding the anatomy and future implications of associated gynecologic anomalies has helped clinicians define the Mullerian anatomy and better plan for puberty, menstruation, sexual function, and future obstetric potential [33,34]. Collaboration with gynecologists has led to recognition of the benefits and drawbacks of early vaginal reconstruction, which become most obvious with long-term care as patients progress through puberty and early adulthood. Emerging trends to avoid bowel vagina reconstructions, delay definitive gynecologic reconstruction when possible, and an emphasis on patient engagement in reconstruction planning hold promise for improved outcomes and patient satisfaction.

### 6.5. Continence Potential

Knowing the anatomic subtype of anorectal malformation along with the quality of the sacrum and the spine allows for an understanding of future continence, the key question on parents’ and their family’s minds. These factors, when considered, have improved the clinician’s ability to explain things to families even in the newborn period [35]. For patients with Hirschsprung disease, surgery that preserves the anal canal and the sphincter mechanism should allow for voluntary bowel movements in the future. Finally, understanding the impact of spinal disorders, given the spectrum of the effect of spina bifida on bowel and urinary control, has led to a better understanding of patients’ quality-of-life challenges.

### 6.6. Newborn Surgical Interventions

Neonatal surgery with the option of a primary pull-through procedure without an initial stoma is an important advance for both ARM and HD. For the patients with an anorectal malformation, this must be balanced with the requirement that the surgeon knows exactly where the distal rectum is located. There is no good way to know this anatomic fact unless there is a visible fistula, so a distal colostogram is needed, which of course requires a colostomy. In most females, except for those with a cloaca, a primary operation without the need for a stoma, even in the newborn period, is possible. This decision must be based on the surgeon’s experience and the clinical circumstances in which they find themselves [34,35,36]. In the case of Hirschsprung disease, a primary repair is usually possible, either in the newborn period or delayed for several months with the patient carrying out daily colonic irrigations at home. The procedure is dependent on good-quality pathology, which is not available in many parts of the world [37].

### 6.7. Defining the Anatomy

**Figure 4:**
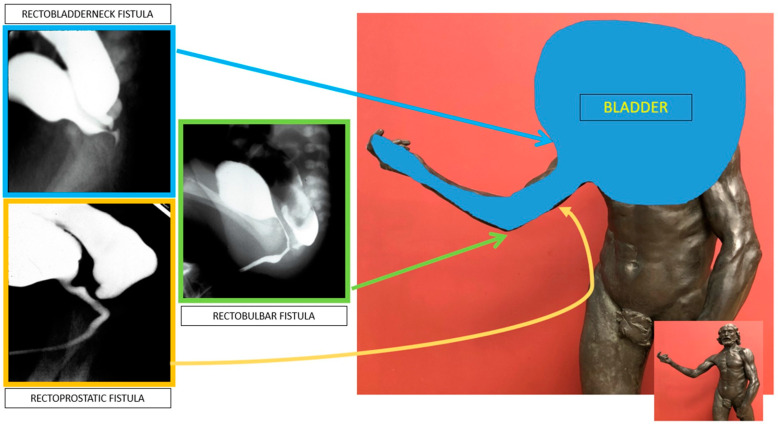
Defining the anatomy for rectourethral fistulae utilizing the analogy of an arm to represent specific anatomic locations.

The collaborative model has led to a protocolized description of the anatomic options for patients with anorectal malformations. The anatomy influences the operative approach chosen and the clinical outcomes expected [Figure 4] [38]. A distal colostogram is vital for defining the location of the rectourethral fistula and the rectum for surgical planning of either a laparoscopic approach or a perineal (posterior sagittal) approach to find the rectum, thereby avoiding injury to the urethra. For Hirschsprung disease, a good contrast study helps the surgeon predict the location of the expected transition zone and properly plan the pull-through.

### 6.8. Minimally Invasive Approaches

A minimally invasive approach to patients with both anorectal malformations [39] and Hirschsprung disease has improved care dramatically. For patients with an anorectal malformation, choosing a laparoscopic approach vs. a posterior sagittal-only approach depends on where the rectum is located. If the rectum is found below the pubococcygeal (PC) line, it is reachable from a posterior sagittal incision and laparoscopy is not needed. If the rectum lies above this line, laparoscopy is the ideal approach. This key decision prevents significant morbidity, particularly the avoidance of a remnant of the original fistula (ROOF) which can occur if the rectum is too low to approach laparoscopically [40]. If a rectum that is too high is approached from a posterior sagittal direction, injury to the urinary tract can occur. In HD pull-through cases, laparoscopy can avoid an abdominal incision and limit the stretching of the sphincters, which can occur during transanal rectal dissection [41].

### 6.9. Cloaca Management

**Figure 5:**
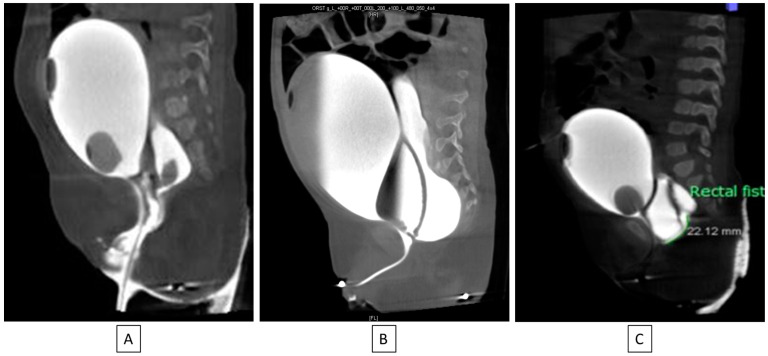
If urethral length is greater than 1.5 cm (**A**,**B**), a total urogenital mobilization can be performed; if it is less than 1.5 cm (**C**), the cloacal repair will need a urogenital separation [40].

The original cloacal repairs described by Hendren all involved separating the vagina from the urinary tract and reconstructing a neourethra. With the major advance of total urogenital mobilization (TUM), the vagina and urinary tract could be brought down as a unit. Thanks to collaborative teamwork between colorectal and urologic surgery, it became clear that the TUM procedure was only appropriate for a patient with a short common channel (<3 cm) and a long urethra (>1.5 cm). Understanding this distinction is critical to recreating the best possible anatomy for the child and minimizing unexpected changes to surgical plans intraoperatively. For example, if a TUM is performed for a patient with a short urethra (less than 1.5 cm), this would lead to an anatomic situation whereby the bladder neck is left too close to the perineum and below the urogenital diaphragm, leading to urinary leakage. On the other hand, if a TUM is performed in the case of a cloaca with a high confluence (long common channel) and the surgeon finds that the UG sinus does not reach the perineum, then a urogenital separation would be required. Since the anterior urethra would already have been dissected during the TUM, this maneuver can lead to urethral loss. Therefore, it is vital to know in advance the common channel and urethral lengths, which has been made possible by precision cystoscopy and cloacogram imaging [42,43] [Figure 5].

## 7. Postoperative Complications for Anorectal Malformation and Hirschsprung Disease

**Figure 6:**
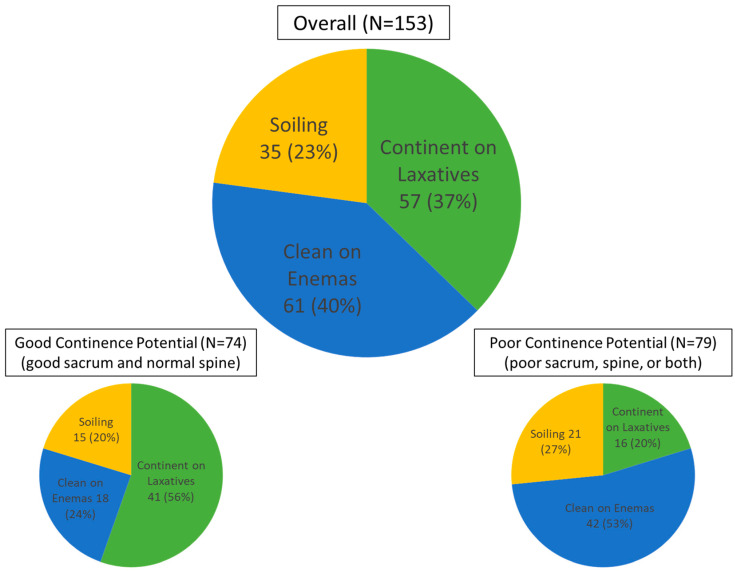
Results following redo for ARM patients with anal mislocation, stricture, prolapse, or ROOF [41].

A number of significant complications are associated with colorectal surgery for ARM and HD. The explanation for this relates to the timing of the surgical misadventure and the realization that one has occurred. Colorectal surgery is unique in this way because, for example, following a PSARP, if the surgeon completes the operation but inadvertently places the anus in the wrong place relative to the sphincters, that mistake would only be realized years later when the child attempts to potty train. If that surgeon recognizes their error 4 years after the operation, it would be very difficult to learn from this technical error and improve their technique. In most of surgery, a complication is recognized much closer to the actual operation. Reoperations for problems such as this, as well as stricture, rectal prolapse, or a remnant of the original fistula (ROOF), may be required. Obviously, it is ideal to avoid the complication in the first place, but if the anatomy is restored with a reoperation, patients can largely go on to achieve fecal continence [44] [Figure 6]. A redo of surgery for HD may be required for a retained transition zone, stricture, retained cuff, dysfunctional Duhamel pouch, or a twist. Once complete, the new anatomy improves flow and can resolve the obstructive symptoms the patient was suffering from [45]. Injured sphincters in HD at the initial pull-through can be repaired using a sphincter reconstruction, which can restore continence [46].

## 8. Innovations in Surgical Technique to Minimize Complications and Improve Outcomes

Educating surgeons on avoiding key dangers and pitfalls in surgical care should be the main focus of innovations in surgical techniques. For patients with ARM, there have been recent innovations with the technique for repairing perineal fistulas. The new technique includes a posterior rectal mobilization with avoidance of an anterior wall dissection, which avoids any periurethral or posterior vaginal wall dissection [47]. There is also a similar technique described for female patients with recto-vestibular fistulas which includes a modification of the traditional PSARP, which avoids any perineal body incision and can, therefore, avoid the feared complication of perineal body dehiscence [48].

## 9. Management of Functional Constipation, Colonic Dysmotility, and Fecal Incontinence

The causes of soiling are myriad, and the care of these patients requires understanding when fecal incontinence should be anticipated as well as the surgical options available to help a patient achieve continence [49]. Bowel management programs and their techniques have dramatically improved many patients’ quality of life and require an institutional commitment to a nursing team [50,51,52,53].

A major advance from the collaborative model has been an improved understanding of motility disorders. Colorectal surgeons working in conjunction with gastrointestinal motility/endoscopic experts has allowed for clarification on when to use laxatives (medical treatment) and when to use retrograde/antegrade enemas (mechanical treatment). The GI/motility/endoscopy team is a key component of the collaborative model. All providers need to agree on what is meant by a “failure of medical management” to understand how evaluate a patient’s anal sphincters, pelvic floor, and colonic motility, and to know when to offer an intervention. Collaborative evaluation algorithms lead to more efficient and accurate diagnoses of underlying causes of constipation, allowing for targeted intervention strategies [54,55]. These interventions include pelvic floor physical therapy, botulinum toxin for internal and external anal sphincter dysfunction, antegrade access for colonic flushes, and surgical removal of a dysmotile colonic segment [56]. For example, our program performs anorectal manometry in conjunction with the GI/motility team to efficiently make an accurate diagnosis and treatment. If there is no recto-ano inhibitory reflex, a rectal biopsy is performed to rule out Hirschsprung disease. If there is evidence of elevated or non-relaxing sphincters, botulinum toxin therapy can also be administered at the same time. Furthermore, a recent article that was made possible by this vital collaboration between GI and surgery showed that antegrade enemas work in the vast majority of patients, which has made segmental colonic resections only rarely necessary [57].

## 10. Colorectal and Urology Collaboration

When colorectal surgery and urology work together, both systems can be managed in parallel. For example, if a bladder augmentation were to be needed, using a segment of colon for this purpose can improve colonic emptying while also enhancing bladder capacity during the same operation. Similarly, the appendix can be shared and be used to become both the Malone ACE and the Mitrofanoff [58]. A study of this sort of proactive planning showed a reduction in the numbers of days in the hospital and the total number of procedures that these patients need [59].

## 11. Transition of Care to Adulthood

An important trend is to plan care for when a pediatric patient becomes an adult. The fields of cardiology and pulmonology have achieved this well with transition programs for congenital heart disease and cystic fibrosis. Similarly, colorectal care needs to expand efforts to create a transition plan for pediatric patients as they enter adulthood [60,61,62].

## 12. Science: Tissue Engineering and Genetics

The collaborative model extends to basic science, and clinicians working with scientists are bringing the “bench to the bedside”. Tissue engineering is poised to revolutionize the field. Soon, gynecologic reconstruction could depend on a tissue-engineered segment of vagina, produced by the patient’s own stem cells [63]. The genetics of anorectal malformations and Hirschsprung disease are being pursued, which will impact parental counseling and treatment options [64,65].

## 13. Consortiums to Enhance Data Collection and Outcomes

Routine, multi-institutional collection and tracking of complication and outcomes is difficult to complete and rare in medicine; however, if successfully accomplished, it can allow for real-time protocol adjustments and improvements in results [66]. For example, if a program routinely tracks wound infections, surgical site infections can be reduced by adopting a GI bundle and changing preoperative antibiotics [67]. The Pediatric Colorectal and Pelvic Learning Consortium (PCPLC), www.pcplc.org, tracks data across multiple institutions [68], which has led to improvements in patient care not achievable by a single institution. When a new protocol is developed, the new idea can be rapidly disseminated across multiple institutions.

## 14. Care in Under-Resourced Areas

Advanced colorectal care is lacking in many parts of the world faced with resource-limited settings. The unique challenges clinicians in these environments face must be understood, and creative solutions should be developed. For example, in regions without the support of pediatric pathology, surgeons will open a colostomy in the dilated portion of the colon for children with HD. They will then pull through that colostomy once it has demonstrated function in vivo. Another example of these creative solutions is the creation of larger-than-anticipated anoplasties in patients with ARM to avoid stricture in patients who may have a lack of follow up due to their particular resources [69].

All previous discussions to this point have been written to help facilitate a collaborative program serving the colorectal and pelvic reconstructive surgical patient. As noted, a high volume of patients allows for a robust database upon which to extract outcome data and shape evidence-based care. With such resources comes a responsibility to educate other providers across the world in producing more qualified surgeons and nurses to care for these patients, regardless of where they might live. Most hospitals do not have the financial resources to support such work. In the developing world, pediatric surgical caregivers are in short supply, and advanced surgical techniques to correct relatively common pediatric congenital colorectal anomalies are often lacking. Colorectal Team Overseas (https://ctoverseas.org/) was formed to reach out and bring this education and training to medical doctors, surgeons, and nurses all over the world so they can care for patients in their areas who are in desperate need of this specialized care. From the hospital’s perspective, encouraging their surgeons and nurses to participate in such trips establishes relationships with these foreign teams as well as their governments. These forged relationships foster trust and can on occasion lead to a pattern of referral to the established collaborative centers when these foreign surgeons find themselves unable to provide such a high level of care.

## 15. Conclusions

The complexity of the care of the colorectal and pelvic reconstruction patient requires an organized approach to bring order to the currently somewhat haphazard state of affairs. We need to strive to make the patient experience like the building of a house, a project for which the collaborative model is so obviously needed. A recent quotation about bringing order where none appears to exist is reproduced here:

*“A” must come before “B”, which must come before “C”, everybody knows that. But what if the Millercamp’s of this world did not have to sit next to the Millerchip’s when it comes to seating arrangements? Can Pat Zawatsky be called before Jack Aaronson when the teacher is taking attendance? Do those 26 letters that make up all the dialogue, signs, thoughts, books, and titles in the English-speaking department of the world need their specific spots in line? Everyone can sing you the well-known jingle from A to Z, but not many people can tell you why the alphabet is the way it is.*

*For almost as long as humans have had the English language, they have had the alphabet. The good ole ABCs. However, the alphabet represents the human need for order and stability. I believe that the same thinking that went into the construct of time and even government went into the alphabet. Justifiably, lack of order leads to chaos. Knife-throwing, gun-shooting chaos, in the case of lack of governmental order. Listen to me when I tell you that there is absolutely no reason that the alphabet is arranged the way that it is. Moreover, the alphabet is simply a product of human nature and how it leads people to establish order for things that do not require it.*

*Now I know this sounds crazy, but bear with me. Only if you really peel away the layers of the alphabet will you find the true weight it carries. People organized the letters of our speech into a specific order simply because there wasn’t already one. Questioning this order will enlighten you on the true meaning of it. Really dig deep into the meaning behind the social construct that is the alphabet. Short and sweet as it may be, the order of the ABCs are much less than meets the eye. There is no reason that “J” should fall before “K!” Understand this. Very important as order is, it is only a result of human nature. What’s next? X-rays become independent of Xylophones in children’s books of ABC’s?*

*You know what the best part is? Zero chance you even noticed that each sentence in this essay is in alphabetical order [70].*

Sir Denis Browne, renowned pediatric surgeon in the United Kingdom, said “The aim of pediatric surgery is to set a standard, not to seek a monopoly”. It is this mission that caregivers who commit to helping children with colorectal and pelvic problems embark on, seeking a high standard by understanding the daily struggle it takes to deliver on the goal of improving a patients’ quality of life. This struggle can win the day when a collaborative care model focused on collaboration and good outcomes is applied. Patients deserve this level of care and will drive this change once the benefits are realized.

## Figures and Tables

**Table 1:** Example list of care provider and other team members that comprise a comprehensive pediatric colorectal surgery program.

Example List of Care Providers on the Colorectal Team
Colorectal surgery
General Surgery
Urology
Anesthesiology
Gastrointestinal Motility
Neurosurgery
Orthopedics
Cardiology
Genetics
Nephrology
Radiology
Interventional Radiology
Psychology
Advanced Practice Providers
**Other providers/business team**
Nursing
Program Director
Nutritionist
Administrative personnel and program specialists
Social Workers
Child life specialist
Care coordinators (schedulers)
Pre-authorization/Insurance specialists
Business Manager
Research Coordinators
Pelvic floor physical therapists
Psychologists

## Data Availability

Not applicable.
